# Soil microbial and nutrient dynamics under different sowings environment of Indian mustard (*Brassica juncea* L.) in rice based cropping system

**DOI:** 10.1038/s41598-021-84742-4

**Published:** 2021-03-05

**Authors:** Sunil Kumar, Ram Swaroop Meena, Rakesh Kumar Singh, Tariq Muhammad Munir, Rahul Datta, Subhan Danish, Gulab Singh Yadav, Sandeep Kumar

**Affiliations:** 1 Department of Agronomy, Institute of Agricultural Sciences, Banaras Hindu University, Varanasi, 221005 India; 2 Publication Unit, Directorate, Indian Agricultural Research Institute, New Delhi, 110012 India; 3 Department of Plant Pathology and Mycology, Institute of Agricultural Sciences, Banaras Hindu University, Varanasi, 221005 India; 4 Department of Geography, University of Calgary, 2500 University Dr. NW, Calgary, AB T2N 1N4 Canada; 5 Department of Agrochemistry, Soil Science, Microbiology and Plant Nutrition, Faculty of Agrisciences, Mendel University in Brno, Zemedelska, Brno, 61300 Czech Republic; 6 Hainan Key Laboratory for Sustainable Utilization of Tropical Bioresource, College of Tropical Crops, Hainan University, Haikou, 570228 China; 7 Division of Agronomy, ICAR-Indian Agricultural Research Institute, Pusa, New Delhi, 110012 India; 8 ICAR-Indian Institute of Pulses Research, Regional Station, Phanda, Bhopal, 462030 India; 9 Present Address: Department of Geography and Planning, University of Saskatchewan, Saskatoon, SK S7N 5C8 Canada

**Keywords:** Soil microbiology, Plant symbiosis, Mass spectrometry, Microbiology techniques

## Abstract

Farmers are not growing diversified crops and applying huge amounts of agrochemicals and imbalanced fertilizers in the rice-wheat cropping system (RWCS), since the 1960s. The objective of this study was to evaluate the microbial and nutrient dynamics in Indian mustard (*Brassica juncea L.*) under various sowing environments and nutrient sources during *Rabi* season (October–March), 2015–2016. The experiment was laid out in the split-plot design with three sowing dates in main-plots, and eight nutrient sources in sub-plots. The maximum bacteria, fungi, and actinomycetes population, soil microbial biomass carbon (SMBC), dehydrogenase activities, and available nitrogen, phosphorus, potassium, and sulphur (NPKS) were recorded on November 17 sown crop, and the lowest was observed on December 7 sowing during both the years, and in the pooled analysis. Furthermore, applied nutrient sources, highest bacteria, fungi, and actinomycetes population, available NPKS, SMBC, and dehydrogenase activity were observed in 75% recommended dose of fertilizers (RDF) + 25% N through pressmud (PM) + *Azotobacto* + *phosphorus solubilizing bacteria* (PSB) than other nutrient sources. In conclusion, high demand and cost of chemical fertilizers can be replaced by 25% amount easily and locally available organic manures like PM compost to sustain the soil health and crop productivity. It will be helpful to restore the soil biodiversity in the RWCS and provide a roadmap for the researchers, government planners, and policymakers for the use of PM as a source of organic matter and nutrients.

## Introduction

In India, rice (*Oryza sativa* L.)-wheat (*Triticum aestivum* L*.*) cropping system (RWCS) is dominant and covering ~ 12.0 m ha area, it is producing about 23% of the total food grains of the country^1^. Intensive RWCS is continuing since the1960s after green Revolution (GR); the result is that the applied inputs use efficiency (IUE) and system productivity is harshly declined^1^. In the GR, intensive use of fertilizers, irrigation water, herbicides, etc*.* helped in boosting crop production. Contrary, during that period, the overexploitation of natural resources (NRs) was also increased, and the nutrient use efficiency (NUE) was decreased continuously^2^. In the RWCS fertilizer (NPK) consumption is 180 kg ha^−1^ higher than India’s average fertilizer consumption (130 kg ha^−1^), and increases the cultivation cost, soil, water, and environmental pollution^3^. Even after a lot of input application wheat yield is declining up to a critical level, it is because due to declining the system productivity, declining in resource use efficiency (RUE) and intensive land cultivation, lack of alternate cropping system, injudicious use of inputs, losses in the SOC pool, and low application of the organic manures (OM), continuously avoidance of biofertilizers, and late wheat sowing is suffering from the terminal heat at the time of maturity^4^. Hence, there is an urgent need to select a suitable crop for diversification in the RWCS dominated area in the developing world with a low input response and avoid a terminal heat effect for better productivity.

In *Rabi* season wheat crop can be replaced with a suitable alternate crop in the RWCS, example is the Indian mustard to sustain the crop and soil productivity. It may maintain the nutrient balance in the cropping systems. Diversification of the system with Indian mustard may prove a beneficial tool to maintain soil health, increase crop and soil productivity, RUE, and farmers' income by decreasing the cost of cultivation^5^. The Indian mustard is a highly responsive crop to the inputs under varied climatic conditions in the RWCS^6^. Indian mustard is one of the important sources of edible oil (2.58 million tons), and playing a key role in the nutritional security^7^. The average yield of Indian mustard is 3.68 q ha^−1^ in 1950–51 and the present average yield is 13.97 q ha^−1^ in India^3^. Thus, Indian mustard is a novel option to diversify the RWCS as a rice-mustard cropping system (RMCS). It will help to enhance the IUE, manage the degraded soil, and improves system productivity.

Rainfall and temperature pattern have moderately changed from the last few decades, and this changing pattern will be continuing in future^8^. To replace the wheat crop with Indian mustard, it is a need to fix the optimum time of sowing for Indian mustard. While due to the changing pattern of rainfall and temperature, it is essential to shift the sowing period to a new optimum time. Then, it will lead to capturing the full potential of the particular crop and variety. In India, Indian mustard is a very important crop that needs the know the optimum time of sowing otherwise, the crop will suffer in terms of morphological, physiological, and biochemical changes and insect-pest attack^1,9^. Sowing time also positively regulates the soil biological properties and processes (microbial and enzymatic activity) and nutrients available to the growing crop^10,11^.

Continuous and long term intensive nutrient mining from the soil leads to cultivated land degradation, while intensive application of chemical fertilizers also leads the soil to degradation due to the accumulation of salts in the rhizosphere^10^. Applications of organic amendment with chemical fertilizers are enhances the labile microbial biomass, nutrient availability, and improve soil properties^12,13^. Soil microorganisms (SMO) called a “biological engine of the earth” is a very important part of the soil ecosystem, and helps in maintaining soil quality^9,14,15^. Healthy soil is estimated to contain earthworms’ 1000 kg ha^−1^, fungi 2700 kg ha^−1^, bacteria 1700 kg ha^−1^, protozoa150 kg ha^−1^ and arthropods 1000 kg ha^−1^ and other small animals^16^. Microbial diversity is directly related to the crops and soil ecosystem services^17,18^. Moreover, soil health has deteriorated for the reason that continuously decreasing SMBC in soils, and rapid decomposition of SOM, and reduction the microbial population in the soil as a result of high temperature, high oxidation of carbon, and drier climate year by year^19^. SOC pool decides the amount of SMBC and their activity in the soil. In many soils, higher microbial activity reduces the fixation of nutrients in the soil and increases the NUE. Thus, it is considered that the microbes help to storage the nutrients in soil systems. Healthy soil is characterized by high microbial and enzymatic activity and helps to sustain the quality of soil, cropping, and environmental ecosystem^1^.

In India, Pressmud (PM) is the waste available from sugar mills in high amounts (8–10 million tons) every year, which can be used as an OM to balance the crop nutrition^19,20^. PM has high nutrient value and it can release the macro and micronutrients required by crop^21,22^. It is an effective soil amendment, and it has value to add with the biofertilizers to restore the soil fertility status. The application of biofertilizers to enhance the nutrient levels of soil to support plant growth and productivity in the agricultural field is a common practice used by the farmers^23,24^.

The novelty of the experiment is the utilization of sugar mill waste (PM), which integrated with RDF and biofertilizers with different sowing dates to supply balanced nutrition to growing crops and sustain the productivity of the system. PM compost is low cost in nature and easily available in sugarcane growing areas, which may be the best alternative of organic matter to farmyard manure (FYM). The current study was conducted with objectives: (1) to find out the effect of sowing dates and nutrient sources on microbial dynamics in the diversified cropping system, and (2) to find the effect of sowing dates and integrated nutrient sources on available nutrients i.e., NPKS dynamics after crop harvest. It is hypothesized that sowing dates and nutrients management can positively influence the microbial dynamics of the rhizosphere.

## Results

### Effect of sowing dates

The perusal of the data (Tables 1, 2, 3) reveals that on November 7 sown crop, the maximum bacterial population (28.25% at 45 DAS, 48.46% at 90 DAS, and 42.85% at harvest stage), fungal population (24.05% at 45 DAS, 42.95% at 90 DAS, and 37.43% at harvest stage), and actinomycetes population (31.01% at 45 DAS, 46.07% at 90 DAS and 38.93% at harvest stage) were increased over November 27 sown crop on the pooled basis. The lowest bacterial population (19.18 at 45 DAS, 32.44 at 90 DAS and 28.76 × 10^7^ CFU g^−1^ soil at harvest stage), fungi population (16.43 at 45 DAS, 28.83 at 90 DAS and 25.21 × 10^7^ CFU g^−1^ soil at harvest stage) and actinomycetes population (20.99 at 45 DAS, 30.87 at 90 DAS, and 26.19 × 10^6^ CFU g^−1^ soil at harvest stage) were observed on December 7 sowing on the pooled basis. Among the sowing dates, the maximum dehydrogenase activity in Table 4 (48.66 µg TPF g^−1^ soil day^−1^) was recorded on November 17 sown crop followed by November 27 (47.02 µg TPF g^−1^ soil day^−1^), and lowest dehydrogenase activity (44.94 µg TPF g^−1^ soil day^−1^) was observed on December 7 sowing in the pooled analysis.

**Table 1:** Effect of sowing dates and nutrient sources on bacterial population (× 10^7^ CFU g^−1^ soil) of Indian mustard.

Treatment	45 DAS			90 DAS			At harvest		
Sowing dates	2015–16	2016–17	Pooled	2015–16	2016–17	Pooled	2015–16	2016–17	Pooled
November 17	29.91	28.59	29.25	51.41	47.52	49.46	48.38	39.33	43.85
November 27	24.43	23.35	23.89	41.98	38.81	40.40	39.51	32.12	35.81
December 7	19.61	18.75	19.18	33.71	31.16	32.44	31.73	25.79	28.76
SEm ±	0.37	0.35	0.26	0.64	0.59	0.43	0.60	0.49	0.39
CD (*p* = 0.05)	1.45	1.39	0.83	2.50	2.31	1.41	2.35	1.91	1.26
**Nutrients sources**									
Control	12.07	11.54	11.80	20.74	19.17	19.96	19.52	15.87	17.69
100% RDF (NPKS)*	21.19	20.26	20.73	36.43	33.67	35.05	34.28	27.87	31.08
100% RDF (NPKS) + *Azotobactor*	24.66	23.58	24.12	42.39	39.18	40.79	39.89	32.43	36.16
100% RDF (NPKS) + PSB	24.92	23.82	24.37	42.83	39.59	41.21	40.31	32.76	36.53
100% RDF (NPKS) + *Azotobactor* + PSB	26.27	25.12	25.70	45.16	41.74	43.45	42.50	34.55	38.52
75% RDF + 25% N through PM + *Azotobactor*	28.36	27.11	27.73	48.74	45.05	46.90	45.87	37.29	41.58
75% RDF + 25% N through PM + PSB	28.75	27.48	28.12	49.42	45.68	47.55	46.51	37.81	42.16
75% RDF + 25% N through PM + *Azotobactor* + PSB	30.97	29.61	30.29	53.24	49.21	51.22	50.10	40.73	45.41
SEm ±	0.15	0.14	0.10	0.25	0.23	0.17	0.24	0.19	0.15
CD (*p* = 0.05)	0.42	0.40	0.29	0.72	0.67	0.48	0.68	0.55	0.43

RDF, Recommended dose of fertilizers; NPKS, Nitrogen, Phosphorus, Potassium and Sulphur; PSB, Phosphorus solubilizing bacteria; PM, Pressmud.

*RDF@100–50-50–40 NPKS kg ha^−1.^

**Table 2:** Effect of sowing dates and nutrient sources on fungi (× 10^5^ CFU g^−1^ soil) population of Indian mustard.

Treatment	45 DAS			90 DAS			At harvest		
Sowing dates	2015–16	2016–17	Pooled	2015–16	2016–17	Pooled	2015–16	2016–17	Pooled
November 17	26.53	23.57	25.05	46.09	41.82	43.95	40.12	36.75	38.43
November 27	21.67	19.25	20.46	37.64	34.15	35.90	32.77	30.01	31.39
December 7	17.40	15.46	16.43	30.23	27.43	28.83	26.31	24.10	25.21
SEm ±	0.33	0.29	0.22	0.57	0.52	0.39	0.50	0.45	0.34
CD (*p* = 0.05)	1.29	1.14	0.72	2.24	2.03	1.26	1.95	1.78	1.10
**Nutrients sources**									
Control	10.70	9.51	10.11	18.60	16.87	17.73	16.19	14.83	15.51
100% RDF (NPKS)*	18.80	16.70	17.75	32.66	29.64	31.15	28.43	26.04	27.24
100% RDF (NPKS) + *Azotobactor*	21.88	19.43	20.66	38.00	34.48	36.24	33.08	30.30	31.69
100% RDF (NPKS) + PSB	22.10	19.64	20.87	38.40	34.84	36.62	33.42	30.61	32.02
100% RDF (NPKS) + *Azotobactor* + PSB	23.30	20.70	22.00	40.49	36.74	38.61	35.25	32.28	33.76
75% RDF + 25% N through PM + *Azotobactor*	25.15	22.35	23.75	43.70	39.65	41.67	38.04	34.84	36.44
75% RDF + 25% N through PM + PSB	25.50	22.66	24.08	44.31	40.20	42.25	38.57	35.33	36.95
75% RDF + 25% N through PM + *Azotobactor* + PSB	27.47	24.41	25.94	47.73	43.31	45.52	41.55	38.06	39.80
SEm ±	0.13	0.12	0.09	0.23	0.21	0.15	0.20	0.18	0.13
CD (*p* = 0.05)	0.37	0.33	0.25	0.65	0.59	0.43	0.56	0.52	0.38

**Table 3:** Effect of sowing dates and nutrient sources on actinomycetes population (× 10^6^ CFU g^−1^ soil) of Indian mustard.

Treatment	45 DAS			90 DAS			At harvest		
Sowing dates	2015–16	2016–17	Pooled	2015–16	2016–17	Pooled	2015–16	2016–17	Pooled
November 17	32.34	31.68	32.01	49.25	44.88	47.07	44.29	35.58	39.93
November 27	26.41	25.87	26.14	40.22	36.65	38.44	36.17	29.06	32.61
December 7	21.21	20.77	20.99	32.30	29.43	30.87	29.04	23.33	26.19
SEm ±	0.40	0.39	0.28	0.61	0.56	0.41	0.55	0.44	0.35
CD (*p* = 0.05)	1.57	1.54	0.91	2.39	2.18	1.34	2.15	1.73	1.15
**Nutrients sources**									
Control	13.05	12.78	12.91	19.87	18.11	18.99	17.87	14.36	16.11
100% RDF (NPKS)*	22.92	22.45	22.68	34.90	31.80	33.35	31.38	25.21	28.30
100% RDF (NPKS) + *Azotobactor*	26.67	26.12	26.39	40.61	37.01	38.81	36.52	29.34	32.93
100% RDF (NPKS) + PSB	26.95	26.39	26.67	41.03	37.39	39.21	36.90	29.64	33.27
100% RDF (NPKS) + *Azotobactor* + PSB	28.41	27.83	28.12	43.27	39.43	41.35	38.91	31.26	35.08
75% RDF + 25% N through PM + *Azotobactor*	30.67	30.03	30.35	46.70	42.55	44.62	41.99	33.74	37.86
75% RDF + 25% N through PM + PSB	31.09	30.45	30.77	47.35	43.14	45.25	42.57	34.21	38.39
75% RDF + 25% N through PM + *Azotobactor* + PSB	33.49	32.80	33.15	51.00	46.48	48.74	45.86	36.85	41.36
SEm ±	0.16	0.16	0.11	0.24	0.22	0.16	0.22	0.18	0.14
CD (*p* = 0.05)	0.45	0.44	0.31	0.69	0.63	0.46	0.62	0.50	0.39

**Table 4:** Effect of sowing dates and nutrient sources on biological soil properties after harvest of Indian mustard.

*Treatment*	Dehydrogenase activity (µg TPF g^−1^ soil day^−1^)			SMBC (mg kg^−1^ soil)			Organic C(%)		
Sowing dates	2015–16	2016–17	Pooled	2015–16	2016–17	Pooled	2015–16	2016–17	Pooled
November 17	49.70	47.63	48.66	198.10	210.09	204.09	0.474	0.474	0.474
November 27	48.02	46.03	47.02	191.43	203.01	197.22	0.472	0.473	0.473
December 7	45.89	43.98	44.94	182.93	194.00	188.47	0.468	0.468	0.468
SEm ±	0.31	0.30	0.22	1.24	1.31	0.90	0.006	0.006	0.004
CD (*p* = 0.05)	1.22	1.17	0.70	4.86	5.15	2.94	NS	NS	NS
**Nutrients sources**									
Control	44.20	42.36	43.28	176.18	186.84	181.51	0.443	0.444	0.443
100% RDF (NPKS)	45.83	43.92	44.88	182.69	193.74	188.22	0.473	0.473	0.473
100% RDF (NPKS) + *Azotobactor*	46.75	44.80	45.78	186.35	197.62	191.98	0.474	0.475	0.474
100% RDF (NPKS) + PSB	46.87	44.92	45.89	186.83	198.13	192.48	0.475	0.476	0.475
100% RDF (NPKS) + *Azotobactor* + PSB	48.05	46.05	47.05	191.54	203.13	197.33	0.475	0.476	0.475
75% RDF + 25% N through PM + *Azotobactor*	49.59	47.53	48.56	197.67	209.63	203.65	0.476	0.477	0.476
75% RDF + 25% N through PM + PSB	49.88	47.81	48.85	198.84	210.88	204.86	0.477	0.478	0.477
75% RDF + 25% N through PM +	51.80	49.64	50.72	206.47	218.97	212.72	0.479	0.479	0.479
*Azotobactor* + PSB									
SEm ±	0.20	0.19	0.14	0.80	0.85	0.58	0.008	0.009	0.006
CD (*p* = 0.05)	0.57	0.55	0.39	2.28	2.42	1.64	NS	NS	NS

NS, Non Significant.

**Figure 1:**
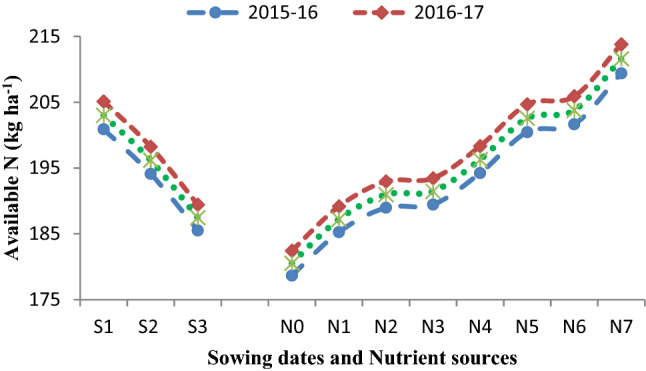
Effect of sowing dates and nutrient sources on available nitrogen (N) after the harvest of Indian mustard.

**Figure 2:**
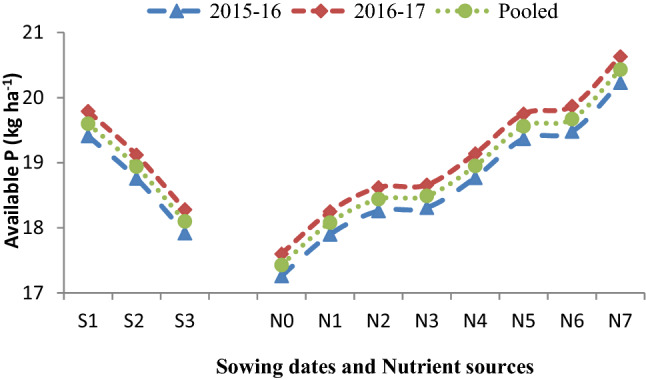
Effect of sowing dates and nutrient sources on available phosphorus (P) after the harvest of Indian mustard.

**Figure 3:**
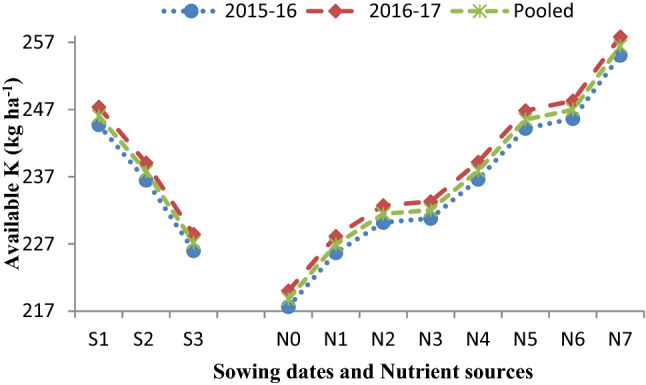
Effect of sowing dates and nutrient sources on available potassium (K) after the harvest of Indian mustard.

**Figure 4:**
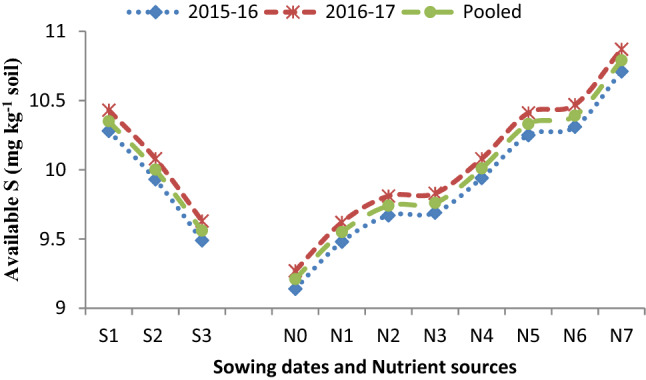
Effect of sowing dates and nutrient sources on available sulfur (S) after the harvest of Indian mustard.

The data (Table 4) was recorded significantly maximum SMBC (204.09 mg kg^−1^ soil) on November 17 sown crop followed by November 27 (197.22 mg kg^−1^ soil), and lowest SMBC (188.47 mg kg^−1^ soil) was observed on December 7 sowing in the pooled analysis. It is apparent from the data (Table 4) found that different sowing dates, as well as nutrient sources, could not bring variation in organic carbon (OC) status of soil after harvest of Indian mustard up to the level of significance. Results (Figs. 1, 2, 3, 4) further showed that among the sowing dates, the maximum available N (203.01 kg ha^−1^), available P (19.60 kg ha^−1^), available K (246.05 kg ha^−1^) and available S (10.28, 10.43, 10.35 mg kg^−1^) was recorded on November 17 sown crop which was 6.83, 0.66, 8.28 kg ha^−1^ and 0.35 mg kg^−1^ higher than November 27sown crop and lowest available NPKS (187.47 kg ha^−1^, 18.10 kg ha^−1^, 227.21 kg ha^−1^ and 9.56 mg kg^−1^, respectively) were observed on December 7 sown crop in the pooled analysis, respectively.

### Effect of nutrient sources

A critical examination of data (Tables 2, 3 and 4) indicated that the entire nutrient sources were significantly increased the microbial population in soil over control during both the years and in the pooled analysis. Among the nutrient sources, the highest bacterial population (30.29 at 45 DAS, 51.22 at 90 DAS, and 50.10 × 10^7^ CFU g^−1^ soil at harvest stage), fungal population (25.94 at 45 DAS, 45.52 at 90 DAS, and 39.80 × 10^7^ CFU g^−1^ soil at harvest stage) and actinomycetes population (33.15 at 45 DAS, 48.74 at 90 DAS, and 41.36 × 10^6^ CFU g^−1^ soil at harvest stage) were observed in 75% RDF + 25% N through PM + *Azotobactor* + PSB than other nutrient sources in the pooled analysis, respectively. While, the application of 75% RDF + 25% N through PM + PSB and 75% RDF + 25% N through PM + *Azotobactor* response on bacterial population (28.12, and 27.73 at 45 DAS, 47.55, and 46.90 at 90 DAS, and 42.16 and 41.58 × 10^7^ CFU g^−1^ soil at harvest stage), fungi population (24.08, and 23.75 at 45 DAS, 42.25, and 41.67 at 90 DAS, and 36.95 and 36.44 × 10^5^ CFU g^−1^ soil at harvest stage) and actinomycetes population (30.77 and 30.35 at 45 DAS, 45.25, and 44.62 at 90 DAS, 38.39, and 37.86 × 10^6^ CFU g^−1^ soil at harvest stage) were found at par to each other in the pooled analysis, respectively. However, the application of 100% RDF + *Azotobactor* + PSB also increased the bacterial population (25.70 at 45 DAS, 43.45 at 90 DAS, and 38.52 × 10^7^ CFU g^−1^ soil at harvest stage), fungi population (22.0 at 45 DAS, 38.61 at 90 DAS, and 33.76 × 10^5^ CFU g^−1^ soil at harvest stage), actinomycetes population (28.12 at 45 DAS, 41.35 at 90 DAS, and 35.08 × 10^6^ CFU g^−1^ soil at harvest stage) in the pooled analysis which was observed higher than 100% RDF + PSB, 100% RDF + *Azotobactor* and control, respectively. Minimum bacterial population (11.80 at 45 DAS, 19.96 at 90 DAS, and 17.69 × 10^7^ CFU g^−1^ soil at harvest stage), fungal population (10.51 at 45 DAS, 17.73 at 90 DAS, and 15.51 × 10^7^ CFU g^−1^ soil at harvest stage) and actinomycetes population (12.91 at 45 DAS, 18.99 at 90 DAS, and 16.11 × 10^6^ CFU g^−1^ soil at harvest stage) was observed in control in the pooled analysis. Results further indicated (Table 5) that the highest dehydrogenase activity (50.72 µg TPF g^−1^ soil day^−1^) was observed in 75% RDF + 25% N through PM + *Azotobactor* + PSB than other nutrient sources in the pooled analysis, respectively. While the application of 75% RDF + 25% N through PM + PSB and 75% RDF + 25% N through a PM + *Azotobactor* response on dehydrogenase activity was found at par with each other. However, the application of 100% RDF + *Azotobactor* + PSB has recorded the dehydrogenase activity (47.05 µg TPF g^−1^ soil day^−1^) higher than the rest of the treatments on a pooled basis. Minimum dehydrogenase activity (43.28 µg TPF g^−1^ soil day^−1^) was observed in control in the pooled analysis. Moreover, results showed in Table 4 that the highest SMBC (212.72 mg kg^−1^ soil) was observed in 75% RDF + 25% N through PM + *Azotobactor* + PSB, which was (31.21 mg kg^−1^ soil) higher than control in the pooled analysis. While, the application of 75% RDF + 25% N through PM + PSB and 75% RDF + 25% N through PM + *Azotobactor* response on SMBC (204.86 and 203.65 mg kg^−1^ soil) found at par with each other on a pooled basis, respectively. However, application of 100% RDF + *Azotobactor* + PSB was observed (Table 5) higher SMBC than 100% RDF + PSB, 100% RDF + *Azotobactor*, 100% RDF, and control during both the years and in the pooled analysis. It is apparent from the data presented in Table 4 that all the nutrient sources could not bring variation in organic C status of soil after harvest of Indian mustard up to the level of significance. A critical examination of the data indicated that among the nutrient sources, the highest available N (211.60 kg ha^−1^), available P (20.43 kg ha^−1^), available K (256.46 kg ha^−1^), and available S (10.79 mg kg^−1^) was observed in 75% RDF + 25% N through PM + *Azotobactor* + PSB in the pooled analysis, respectively than other nutrient sources. While, the application of 75% RDF + 25% N through PM + PSB and 75% RDF + 25% N through PM + *Azotobactor* was found at par with each other, respectively. However, application of 100% RDF + *Azotobactor* + PSB was observed the higher available N (15.74 kg ha^−1^), available P (1.52 kg ha^−1^), available K (19.08 kg ha^−1^), and available S (0.8 mg kg^−1^) than control in the pooled analysis, respectively. Moreover, the 100% RDF + PSB and 100% RDF + *Azotobactor* effect on available NPKS was found at par with each other during both the years and in the pooled analysis, respectively.

**Table 5:** Initial soil chemical and biological properties.

Particulars	2015–16	2016–17	Method employed
	Value		References
Organic C(%)	0.42	0.45	(45)
Available N (kg ha^−1^)	205.7	210.3	(46)
Available P_2_O_5_ (kg ha^−1^)	19.4	21.1	(47)
Available K_2_O (mg kg^−1^)	210.20	219.3	(48)
Available S (mg kg^−1^)	20.78	22.56	(49)
Bacteria 10^7^ colony forming unit (CFU) g^−1^ soil	27	26	(50)
Fungi 10^5^ CFU g^−1^ soil	20	18	(50)
Actinomycetes 10^6^ CFU g^−1^ soil	16	14	(50)
Soil Biomass Carbon (μg g^−1^)	138	141	(51)
Soil dehydrogenase activity(DHA) (ìg TPF g^−1^ soil day^−1^)	120	122	(52)

## Discussion

### Effect of sowing dates

Suitable soil environmental conditions with sustained soil fertility are the key tools that help in catching the full potential of the growing crops in terms of higher growth and production. The data of microbial population and dehydrogenase activity (Tables 1, 2, 3, 4) revealed that different sowing dates had a significant effect on microbial population. Among the sowing dates, the maximum population of bacteria, fungi, actinomycetes, and dehydrogenase activity were recorded on November 17 sown crop during both the growing periods and after harvest of Indian mustard, which significantly differed from other sowing dates. The bacterial population (Table 3) showed a gradual decreased with an increased gap of sowing date from November 17 to December 7. The microbial population peaked during winter followed by spring and autumn, respectively. Tables 1, 2, 3 indicated that the microbial population was significantly increased in the first sown crop. It may be due to the microbial population was significantly influenced by temperature and OM content of the soil, which was found suitable in the first sowing. At the time of harvesting lower bacterial population was observed in the last sowing due to initially very low decomposition of OM in the soil which results in the low microbial population in later stages and also decreases in C food to the microbes^10,25^. Enzymatic activity (Table 4) was found to be higher in the soil of the first sown crop due to soil enzymes providing insights into biogeochemical cycling of C and other nutrients to the microbial functions^16,26^. The data of available nutrients presented in Figs. 1, 2, 3 and 4, revealed that different sowing dates significantly affected the available nutrients after harvest of Indian mustard. Among the sowing dates, the maximum available NPKS and SMBC were recorded on November 17 sown crop in the pooled analysis that may be due to the longer growth period of the crop in which more mineralization of soil organic matter takes place as compared to other sowing dates. First sown crop increased the level of available N (Fig. 1), this may be due to high microbial population in soil (Tables 1, 2, 3) results in high mineralization of organic manure, which improves the physicochemical properties of soil and also improves the availability of the nutrients to the crop to fulfilling the crop requirement and ultimately improved the soil fertility. Available P of soil (Fig. 2) significantly improved with the first sown crop after harvest may be due to the higher rate of mineralization and favourable condition for microbial activity as well as chemical activity^25^. Available K of soil was (Fig. 4) improved with the application of nutrients might be due long growth period providing more opportunities to more mineralization of potassium in soil on first sown crop^20^. The higher available S content in soil (Fig. 4) could be attributed to greater mineralization and release of S as SO_4_^2−^ ions from organic material after its oxidation due to suitable soil temperature and soil health in the first sown crop^27^. Likewise, the increase in the SMBC in soil (Table 4) with first sowing was probably due to the favourable moisture and temperature conditions and increased decomposition of SOM providing more source of utilization for microbes, which was attributed to greater SMBC^16,28^.

### Effect of nutrient sources

The data on microbial population (Tables 2, 3, 4) indicated that the significantly highest population of bacteria, fungi, actinomycetes, and dehydrogenase activity were observed in 75% RDF + 25% N through PM + *Azotobactor* + PSB in the pooled analysis. It may be due to the bacterial population was influenced significantly by PM application with inorganic fertilizers and microbial inoculation, which significantly increased the microbial population over all other nutrient sources. At harvest lower microbial population was observed as compared to the 90 DAS stage of Indian mustard due to decomposition of OM more rapidly at peak growth stages and reduced availability of nutrients compared to the flowering stage of Indian mustard^29,30^. It may be due to the microbial breakdown debris and help to fasten the decomposition process, and enhanced content of total N from the PM, ultimately multiplying very fast. Soil enzymes (dehydrogenase) function as a measurement of the metabolic condition of soil microbes by relating it to the occurrence of feasible microorganisms and their oxidative capability^31^. Data on maximum dehydrogenase activities after harvesting of the crop (Table 3) observed with 75% RDF + 25% N through PM + *Azotobactor* + PSB. It may be due to higher OM supplied with PM, and fast decomposition. The dehydrogenase activity increased with the addition of PM could be attributed to increased microbial activities, which also encourage the dehydrogenase activity^32^.

The data on available nutrients were significantly influenced by nutrient sources. The available N significantly increased after harvest in soil (Fig. 1). It may be due to the mineralization of PM in soil and the initial availability of N through chemical fertilizers and N fixation with *Azotobactor*. The PM is a store house of all the essential plant nutrients required for crop growth. PM application improved the soil environment by improving the physicochemical and biological properties of soil^33^. The availability of most of the essential plant nutrients increased owing to the improvement in pH and the cation exchange capacity (CEC) of soil. The available P content (Fig. 2) of soil increased significantly with the application of 75% RDF + 25% N through PM + *Azotobactor* + PSB might be due to the greater solubilization and mobilization of fixed native soil P by micro-organisms (PSB), vigorous root proliferation and contribution through biomass. The available K content of soil increased significantly with the application of 75% RDF + 25% N through PM + *Azotobactor* + PSB. The buildup of available K in soil under PM application resulted in additional K supplied through it. The solubilizing action of various organic acids released during the decomposition of PM, and it has a better capacity to hold K in the soluble form. The availability of S content (Fig. 4) in soil significantly increased because PM is a good source of S in soil and the slow release nature of PM as OM resulted in higher residual S availability. Blending of 25% N through Pressmud and 75% NPKS through fertilizers could only meet the nutrient demand of the crop resulted in higher uptake and yield of Indian mustard. The SMBC (Table 4) of soil significantly increased with the application of 75% RDF + 25% N through PM + *Azotobactor* + PSB. It may be due to the microbial biomass which depends on the type of OM added, and the organic source being able to supply essential nutrients to soil fauna and to the plants^34,35^. The addition of organic C with the PM application stimulated microbial population and growth thus mineralization of nutrients in the PM^36–38^. Although plants and microbes may at the start compete for nutrients, the organic source supplied adequate nutrients for plants and soil microbes, particularly PM which had high nutrients concentration^39–42^. Further, microbial decomposition could release essential nutrients for plant uptake when the readily available organic C from the PM^43–46^.

## Conclusion

Based on the two years experiment on diversified Indian mustard, it is concluded that the best treatment combination response on the microbial population, enzymatic activities, SMBC and available nutrients after harvest of the crop as follows:

- Diversified Indian mustard sowing in the first fortnight of November sowing is a novel approach in the intensive rice-based cropping system.
- Results reveal that the crop sowing in the first fortnight of November and applied nutrient source as 75% RDF + 25% N through PM + *Azotobactor* + PSB significantly increased all the recorded parameters except organic C content.
- High demand and cost of chemical fertilizers can be replaced 25% amount easily and locally available organic manures like PM compost to sustain the soil health and crop productivity.
- This study will help the scientific society, producers, and policymakers for planning to convert the industrial waste as a nutrient source with bio-inoculants to restore the soil health in RWCS.

## Materials and methods

### Location and climate

A field experiment was carried out during two *rabi* seasons of 2015–2016 and 2016–2017 at the Agricultural Research Farm, Institute of Agricultural Sciences, Banaras Hindu University, Varanasi, situated between 25° 18′ North latitude, 83° 30′ East longitudes and at an altitude of 76.216 m from the mean sea level^47^.

The total rainfall of 61.3 mm was received during the experimentation cropping period of the first year was higher than the second year of the experiment having 52.1 mm. The weekly mean maximum temperature (19 to 41.4 °C and 20.1 to 42.2 °C) and minimum temperature (7.2 to 29.9 °C and 8.2 to 27.3 °C) were recorded during both the years of experimentation, respectively. The average sunshine hours during the 2015–16 (6.43 h) was comparatively higher as compared to the 2016–17 (5.92 h). The texture of experimental soil was sandy clay loam, which was well-drained. Among the major nutrients, the soil was low in available N and P and medium in available K.

### Treatments and treatments and experimental design

The experiment was designed in a split-plot design (SPD) with three replications. In main-plots three sowing dates (November 17, 27 and December 7) and in sub-plots eight nutrient sources (control, 100%RDF (NPKS), 100%RDF + *Azotobactor*, 100%RDF + PSB, 100%RDF + *Azotobactor* + PSB, 75% RDF + 25% N through PM + *Azotobactor*, 75% RDF + 25% N through PM + PSB, 75% RDF + 25% N through PM + *Azotobactor* + PSB) were planned. RDF @100–50-50–40 NPKS kg ha^−1^ is recommended for this region, and urea, Di-ammonium phosphate (DAP), muriate of potash (MOP), and elemental S were used as a source of fertilizers, respectively. The experimentation field was cross ploughing by tractor-drawn cultivator with a rotavator and divided into blocks and plots according to the plan of the layout.

**Figure 5:**
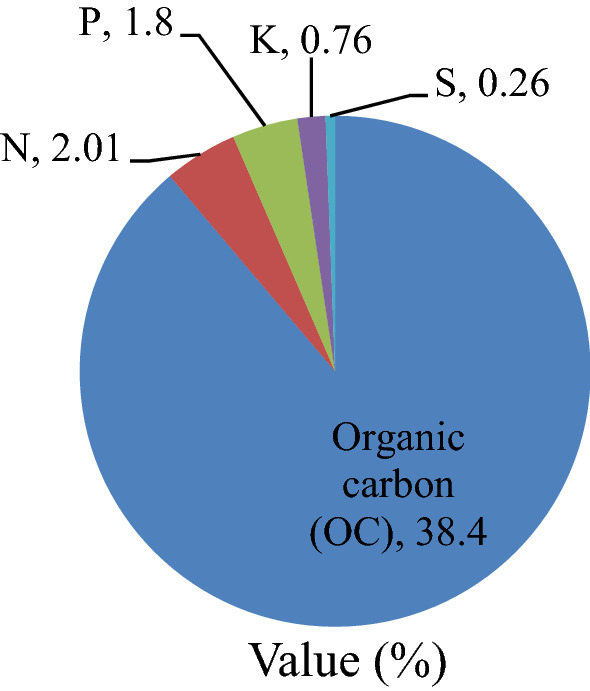
Chemical composition of pressmud (PM) compost.

A full dose of PKS and half dose of N after adjusting with DAP was applied as a basal and the remaining half dose of N was top-dressed at 40 DAS through urea. Applied PM chemical composition is presented in Fig. 5.

In PM compost treatments 25% N was applied through PM compost which was thoroughly mixed in soil one week before sowing and the remaining 75% N was applied through fertilizer. The seed @ 5 kg/ha was used. Biofertilizers *i.e. Azotobacter* and PSB were used for seed treatment as per the standard procedure. The remaining agronomic practices are followed as per the crop requirement**.**

### Soil analysis and recording the observations

Five soil samples were collected from each treatment to assess the nutrient and biological properties of the experimental soil. The samples were brought to the laboratory, processed, and subjected to standard chemical and biological analyses as presented in Table 5.

### Statistical analysis

For calculating the significance between the treatment means and to draw a valid conclusion, statistical tools and techniques were adopted by the appropriate method^48^.

## Abbreviations

CFU: Colony forming unit
CEC: Cation exchange capacity
DAP: Diammonium phosphate
FYM: Farm yard manure
GR: Green revaluation
IUE: Input use efficiency
MOP: Muriate of potash
NUE: Nutrient use efficiency
NRs: Natural resources
NPKS: Nitrogen, phosphorus, potassium and sulphur
OM: Organic matter/organic manure
OC: Organic carbon
PM: Pressmud
PSB: Phosphorus solubilizing bacteria
RDF: Recommended dose of fertilizers
RUE: Resource use efficiency
RWCS: Rice-wheat cropping system
SPD: Split-plot design
SMBC: Soil microbial biomass carbon
SOC: Soil organic carbon
SMO: Soil microorganism
